# Cow’s Milk Protein Allergy: ETAPA Survey on Pediatric Management and Tolerance Acquisition

**DOI:** 10.3390/children12121645

**Published:** 2025-12-03

**Authors:** Juan José Díaz-Martin, Rafael Martín-Masot, Alicia Santamaría-Orleans, Víctor Manuel Navas-López

**Affiliations:** 1 Unit of Pediatric Gastroenterology and Nutrition, Hospital Universitario Central de Asturias, 33011 Oviedo, Spain; diazmjuan@uniovi.es; 2 Pediatric Gastroenterology and Nutrition Unit, Hospital Regional Universitario de Málaga, 29010 Málaga, Spain; rafammgr@uma.es (R.M.-M.); victorm.navas.sspa@juntadeandalucia.es (V.M.N.-L.); 3 Scientific Communication Department, Laboratorios Ordesa, S.L., 08038 Barcelona, Spain

**Keywords:** cow’s milk protein allergy, oral food challenge, pediatric practice, tolerance acquisition

## Abstract

**Highlights:**

**What are the main findings?**

- Spanish pediatricians show substantial variability in the diagnosis and management of cow’s milk protein allergy (CMPA), particularly in oral food challenge use, elimination diet duration, and tolerance monitoring.
- Although many practices follow current guidelines, inconsistent application leads to risks of overdiagnosis, nutritional deficiencies, and delayed tolerance development.

**What are the implications of the main findings?**

- Strengthening professional training, parental education, and standardized diagnostic and reintroduction procedures is crucial to improve safety and promote earlier tolerance in both IgE- and non-IgE-mediated CMPA.
- National pediatric and allergy societies should drive the development of unified diagnostic pathways, tolerance monitoring protocols, and formula recommendations, alongside closer coordination between primary and hospital care. Implementing national policies for routine reassessment and timely reintroduction would align Spanish practice with international standards and improve children’s long-term quality of life.

**Abstract:**

**Background:** Cow’s milk protein allergy (CMPA) is among the most common pediatric food allergies. Understanding tolerance acquisition and diagnostic approaches is critical for optimizing care, particularly in Spain, where regional differences may impact management. This study analyzed real-world practices for determining CMPA oral tolerance acquisition by Spanish pediatricians. **Methods:** A questionnaire was distributed to healthcare providers in primary and hospital pediatric settings across various Spanish provinces. The survey addressed demographic profiles, diagnostic approaches, tolerance acquisition, and dietary modifications. **Results:** Among the 269 health professionals included (mean age 48.3 ± 11.0 years, 62.3% women), most worked in primary care (55.4%), 20.4% in public hospitals, and 17.8% in private practice. Overall, 35.5% routinely referred CMPA cases to specialists. Specific IgE testing (27.9%) and elimination diets (41.3%) were the main diagnostic tools. Diagnostic dairy-exclusion duration varied, mainly in non-IgE cases. Hospital-based diagnostic oral food challenges (OFC) were preferred for suspected IgE-mediated cases (95.7%), while home-based protocols were used for non-IgE suspected cases (80.7%). Guideline adherence for home-based OFC varied by specialty. Tolerance acquisition was evaluated annually by 67.7% of participants, with a mean success rate of 80%. Therapeutic dairy-exclusion duration varied, with 64.7% excluding CMP for 6–12 months. Minimum age for CMP reintroduction was considered dependent on phenotype and severity, with 12 months of age mentioned most frequently (17.1%). **Conclusions:** Despite general alignment with international guidelines, relevant variability exists in CMPA management in Spain. Harmonizing diagnostic and therapeutic practices across specialties and care levels may help standardize care and improve patient outcomes.

## 1. Introduction

Cow’s milk protein allergy (CMPA) is the most prevalent food allergy during infancy and early childhood, typically beginning within the first year of life, and affecting approximately 2% of children under four years of age [1,2,3,4,5].

From a pathophysiological perspective, CMPA can be classified into two main forms: an IgE-mediated type with immediate-onset symptoms (within minutes to less than two hours after ingestion), usually cutaneous or respiratory; and a delayed, non-IgE-mediated type (onset between 2 h and several days post-ingestion), which predominantly presents with gastrointestinal manifestations, including food protein-induced enterocolitis syndrome (FPIES) [3,5,6,7,8].

According to the European Society for Pediatric Gastroenterology, Hepatology and Nutrition (ESPGHAN), diagnosis in both breastfed and formula-fed infants should be based on a short elimination diet followed by a mandatory oral food challenge (OFC), except in cases presenting with severe allergic symptoms, such as anaphylaxis or markedly elevated CMP specific IgE levels [5]. In terms of therapeutic management, extensively hydrolyzed (casein and/or whey) formulas (eHF) are recommended as first-line nutritional treatment, with amino acid formulas (AAF) reserved for severe or refractory cases; hydrolyzed rice formulas (HRF) may be used as alternatives despite lower evidence [5]. In accordance with the most recent WAO DRACMA 2024 recommendations, when choosing a formula for non-breastfed infants with non-IgE-mediated CMPA, “an extensively hydrolyzed (cow’s milk) formula or a hydrolyzed rice formula is suggested as the first option, amino-acid formula as the second option, and soy formula as the third [9]”.

Importantly, the central goal of CMPA management is the acquisition of oral tolerance, which occurs in approximately 85% of children with CMPA before the age of 3 [10]. This is achieved through a process of supervised CMP reintroduction after a period of symptom control. In non-IgE-mediated cases, CMP can often be gradually introduced using structured protocols such as the “milk ladder.” However, in IgE-mediated CMPA, tolerance is more difficult to achieve, and reintroduction requires close monitoring and individualized evaluation of IgE levels [5]. Inadequate diagnostic and management procedures may lead to both over- and under-diagnosis, resulting in unnecessary dietary restrictions and potential nutritional risks that can compromise nutritional status, impair growth, and reduce quality of life [3,5,11].

In real-world clinical settings, the diagnostic-therapeutic pathways of CMPA often involve pediatricians working across multiple healthcare levels, including both primary and hospital settings, within public and private sectors. Therefore, despite the availability of clinical guidelines, substantial variability exists in role assignments, influenced by organizational, geographic, and individual factors [11]. In particular, a national survey among Spanish pediatric gastroenterologists revealed inconsistent application of OFCs and diverse preferences in formula selection, as well as notable gaps in training between professionals and units [11].

In this context, effective coordination between primary and hospital care is essential to ensure the appropriate dietary management of CMPA [11]. The aim of this study was to explore in depth the degree of knowledge and adherence to the latest clinical guidelines and expert consensus documents relevant to the diagnosis and management of CMPA of Spanish pediatricians. Additionally, it aimed to examine the current practices applied by them in assessing the acquisition of oral tolerance, as well as to analyze the specific role of different pediatric specialists in this process for both IgE- and non-IgE-mediated forms.

## 2. Materials and Methods

### 2.1. Study Design and Participants

The Survey on Tolerance Acquisition in Cow’s Milk Protein Allergy in Primary and Hospital Pediatric Care (ETAPA) project was a cross-sectional, observational study, which included pediatricians working in primary care and hospital-based pediatric units across Spain, from both the public and private sectors.

A structured online survey was developed to collect data on clinical practices, guideline knowledge, and roles played by pediatricians in the diagnostic and follow-up of CMPA patients. Pediatricians were invited to participate via personalized emails using a national pediatric database maintained by Laboratorios Ordesa S.L. (Barcelona, Spain), which includes 5187 professionals, representing approximately half of all pediatricians in Spain. Candidates regularly involved in the diagnosis and management of CMPA and who provided informed consent were included. To ensure that the survey reflected real-world clinical practice, no predefined diagnostic definition of CMPA was imposed. Instead, CMPA was operationally defined by the participating pediatricians according to their usual clinical criteria. The questionnaire captured the specific tools used by respondents to establish both diagnostic suspicion and confirmation of CMPA, including clinical history, physical and nutritional assessment, elimination diet, oral food challenge (OFC), specific IgE testing, skin prick testing, and symptom evolution. This approach allowed us to characterize diagnostic practices as they are applied in routine care, rather than restricting responses to guideline-based or study-mandated definitions. No additional inclusion or exclusion criteria were applied. Participation was voluntary and anonymous between January and September 2024.

### 2.2. Study Questionnaire and Data Collection

The questionnaire was developed by a scientific advisory board consisting of pediatric specialists in allergy and gastroenterology, after a comprehensive literature review (Appendix A). It encompassed three main domains: (1) general characteristics of the participating pediatricians and their work settings; (2) diagnostic strategies, referral criteria, and CMP reintroduction approaches; and (3) CMPA management strategies assessed through 12 items focused on criteria and procedures for evaluating oral tolerance and preferences regarding specialized formula use. The questionnaire combined closed-ended and multiple-choice questions, designed to capture real-world clinical approaches to CMPA. Domains two and three included selected items derived from current ESPGHAN guidelines to evaluate adherence to recommended practices and assess the level of agreement or disagreement with evidence-based recommendations.

To evaluate the influence of pediatricians’ specialty (gastroenterologists and pediatric allergologists (G/PA) Vs. General pediatricians) on clinical practices, intergroup analysis was performed for each question.

The survey was administered online and required approximately 10 min to complete. The final version was developed by consensus after a comprehensive review of both national and international CMPA clinical guidelines.

### 2.3. Statistical Analysis

Categorical variables were summarized using absolute frequencies and percentages, while continuous variables were expressed as means and standard deviations (±SD), or as medians and interquartile ranges, depending on their distribution. Associations between categorical variables were analyzed using Fisher’s exact test. For continuous variables, comparisons between groups were performed using Student’s *t*-test or ANOVA, as appropriate. A *p*-value < 0.05 was considered statistically significant.

Given the observational, cross-sectional design and the descriptive aims of the study, only basic inferential analyses (Fisher’s exact test and *t*-test/ANOVA) were conducted. Multivariate analyses and advanced statistical modeling were not performed. All analyses were carried out using IBM SPSS Statistics version 26.0 (IBM Corp., Armonk, NY, USA). Missing values were excluded from percentage calculations when appropriate and were not imputed, except in descriptive summaries.

## 3. Results

### 3.1. General Characteristics of Participants

A total of 269 pediatricians participated in the study (mean age 48.3 ± 11.0 years; 62.3% female). Most respondents worked in the public sector (60.1%), 19.8% in private practice, and 20.2% in both. Urban environments accounted for the majority of work settings (87.8%). Workplaces included primary care centers (55.4%), public hospitals (20.1%), and private clinics (17.8%). Most participants specialized in general pediatrics (71.4%), with smaller groups in pediatric gastroenterology (19.1%) and allergology (4.2%) (Supplementary Table S1).

### 3.2. CMPA Diagnosis

Nearly all respondents (94.8%) relied on a detailed clinical history, including physical examination, nutritional assessment, and dietary history, as the basis for CMPA diagnosis. Reported diagnostic methods included diagnostic elimination diet (41.3%), specific IgE testing (27.9%), oral food challenge (OFC) with infant formula (26.4%), symptom rating scales (25.3%) such as the Cow’s Milk-related Symptom Score (CoMiSS™), and, less commonly, skin prick testing (12.6%) (Supplementary Table S2). Compared with general pediatricians, gastroenterologists and pediatric allergists (G/PA) were more likely to use skin prick tests (*p* = 0.0477) as well as OFC (*p* = 0.0206) in diagnosis (Supplementary Table S3).

Non-IgE-mediated cases were typically associated with gastrointestinal symptoms (e.g., irritability/colic: 92.2%, regurgitation/vomiting: 89.2%, diarrhea: 87.7%, constipation: 84.0%). In contrast, IgE-mediated CMPA presented predominantly with acute allergic symptoms (e.g., anaphylaxis: 92.6%, urticaria: 88.5%, angioedema: 87.7%, wheezing and asthma: 74.0%, and oral allergy syndrome: 71.0%; Supplementary Table S4).

Regarding the diagnostic approach to CMPA, for suspected non-IgE CMPA, 44.3% of respondents performed a diagnostic elimination diet for ≤4 weeks, while 23.5% extended the exclusion for ≤6 weeks. Practices were more heterogeneous in suspected IgE-mediated cases, with only 23.0% restricting exclusion to ≤4 weeks and 37.6% using variable durations (Supplementary Table S5).

After symptom resolution, 48.7% always performed an OFC, except in cases of severe FPIES, to confirm non-IgE suspected CMPA (OFC-D), while 2.7% never performed this step. In case of IgE-mediated suspicion, 10.8% reported routinely conducting an OFC-D. Over 60% answered “rarely” or “never” (Supplementary Table S5). When analyzed by specialty, G/PAs were less consistent than general pediatricians in performing OFC-D to confirm non-IgE CMPA (*p* = 0.0067; Supplementary Table S3).

**Figure 1:**
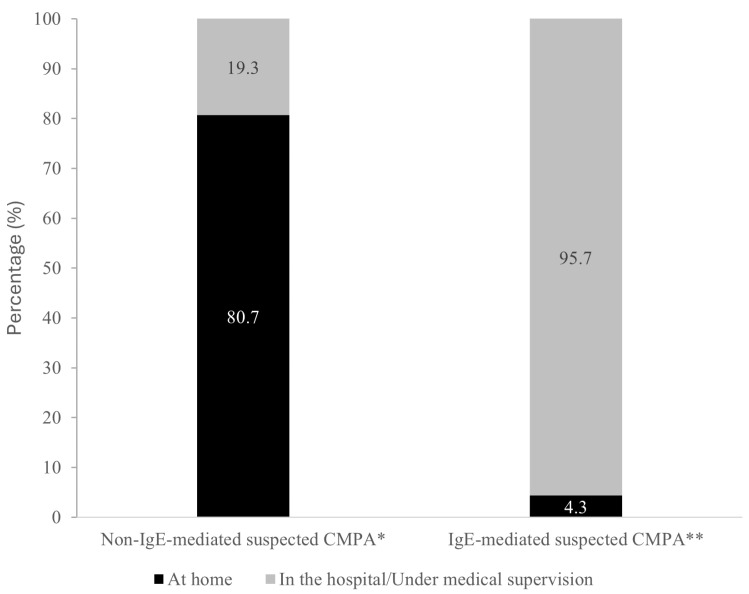
Preferred setting for performing CMPA diagnostic challenges. Total number of valid responses: * N = 264; ** N = 253.

Moreover, the preferred OFC-D setting differed by phenotype. Home-based OFC-D was preferred for non-IgE suspected cases (80.7%), while hospital-based/supervised OFC-D was preferred in IgE suspected ones (95.7%) (Figure 1). Similarly, OFC-D protocols also varied by feeding method and phenotype. Among hypoallergenic formula-fed infants with suspected non-IgE-mediated CMPA, 91.1% of pediatricians advised dairy-free formula replacement with a standard CMP formula. For breastfed non-IgE cases, 90.5% of the respondents advised dairy reintroduction in the maternal diet. In IgE-mediated suspected cases, 67.2% recommended initiating with a very small CMP dose and gradually increasing it (Table 1). By specialty, results showed that G/PAs preferentially followed their own protocols rather than current guidelines (*p* < 0.05) (Supplementary Table S3).

Most pediatricians (89.5%) consistently told families not to introduce new foods during the OFC-D period, with 6.4% giving this recommendation sometimes or rarely/never (1.9%) (Supplementary Table S6).

Following a diagnosis of CMPA, almost half of the surveyed pediatricians (49.8%) reported that the decision to refer to a specialist depends on the individual case. Meanwhile, 35.5% consistently refer cases to specialists, and 14.7% follow the cases exclusively in primary care. By phenotype, in IgE-mediated cases, the majority of pediatricians opted for referral to allergology services (69.3%), while 30.3% chose to refer to pediatric gastroenterology. In contrast, in non-IgE-mediated cases, referrals were predominantly directed to gastroenterology (92.4%), with a minority referring to allergology (6.8%) (Supplementary Table S7). Relevantly, G/PAs were more likely to consider that cases are referred to specialists (*p* < 0.0001), when compared to general pediatricians (Supplementary Table S3).

Overall, clinicians estimated that 70.2% of their annual CMPA caseload was non IgE mediated (85.3% mild/moderate; 14.7% severe), while 27.3% was IgE mediated (80.4% mild/moderate; 19.6% severe) (Supplementary Table S7).

### 3.3. CMPA Follow-Up

Regarding the maintenance of a CMP-free diet, in mild/moderate non-IgE CMPA, 44.4% maintained the exclusion diet for 3–6 months, and 34.2% for 6–12 months. For IgE-mediated CMPA, 51.7% maintained the exclusion diet for 6–12 months, and 35.9% for >12 months. (Supplementary Figure S1A). The data collected further showed that most professionals surveyed (51.4%) believe that the minimum age recommended to maintain a dairy-free diet should be adapted based on the clinical phenotype or severity of the condition; 17.8% favored up to 12 months of age, and 13.5% considered up to 9 months sufficient (Supplementary Figure S1B).

Before dairy reintroduction, 80.2% of pediatricians routinely performed specific IgE or skin prick testing in IgE-mediated cases, whereas pre reintroduction testing was rarely performed in non-IgE-mediated ones (37.2%) (Supplementary Figure S1C). When analyzed by specialty, G/PA more often tested IgE or skin prick before CMP reintroduction in both CMPA phenotypes (*p* < 0.0355) (Supplementary Table S3).

**Figure 2:**
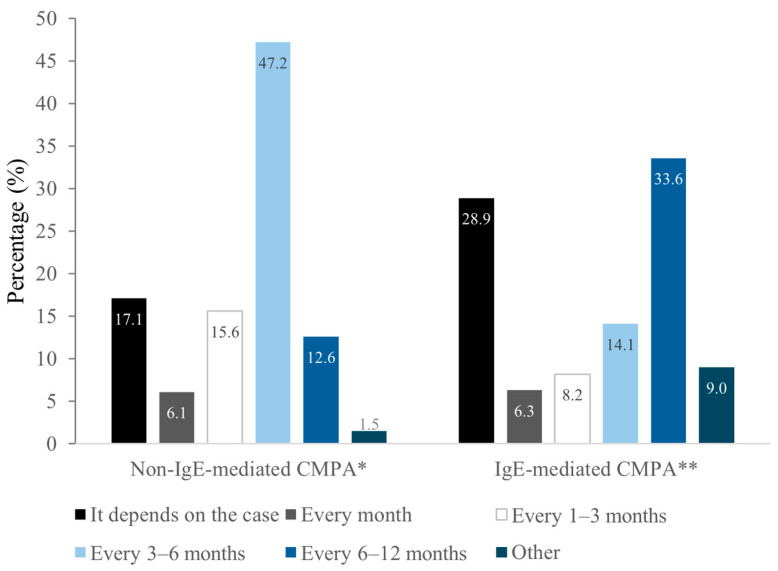
Frequency of tolerance monitoring in CMPA cases. Total number of valid responses: * N = 263; ** N = 256.

Tolerance reassessment intervals differed by phenotype. For non-IgE CMPA, 47.2% of respondents reassessed tolerance every 3–6 months, while 17.1% adjusted the timing based on individual cases. In IgE-mediated cases, 33.6% reassessed every 6–12 months, and 28.9% tailored the frequency to clinical context (Figure 2).

The “milk ladder” was the preferred approach for home tolerance testing (96.6%) in non-IgE-mediated CMPA. Even so, 3.5% of pediatricians followed protocols based on workplace practices or individual clinical judgment (Table 2).

To ensure appropriate implementation of home-based tolerance acquisition tests in mild/moderate non-IgE-mediated CMPA, printed materials were used by 73.8% pediatricians to supplement in-office instructions, while online resources were rarely used (4.9%). In case of a previous OFC with an unfavorable outcome, 72.1% recommended performing the tolerance acquisition test in a hospital under medical supervision; 27.9% still supported home-based testing (Table 2). Overall, 81.7% of pediatricians considered home-based tolerance testing in patients with non-IgE-mediated CMPA “totally” or “mostly” safe when performed according to established guidelines (Supplementary Table S8).

By specialty, G/PA more often supplemented parental guidance on home-based tolerance testing with printed materials (*p* = 0.0131), and recognized home-based tolerance tests as safer (*p* = 0.0307). G/PA further preferred home-based tolerance testing in patients with prior unfavorable OFC (*p* = 0.0000). Finally, their approaches to home-based tolerance testing deviated more from standard “milk ladder” strategies (*p* = 0.0080), when compared to general pediatricians (Supplementary Table S3).

### 3.4. Preferences and Perceptions Regarding Infant Formulas in the Management of CMPA

**Figure 3:**
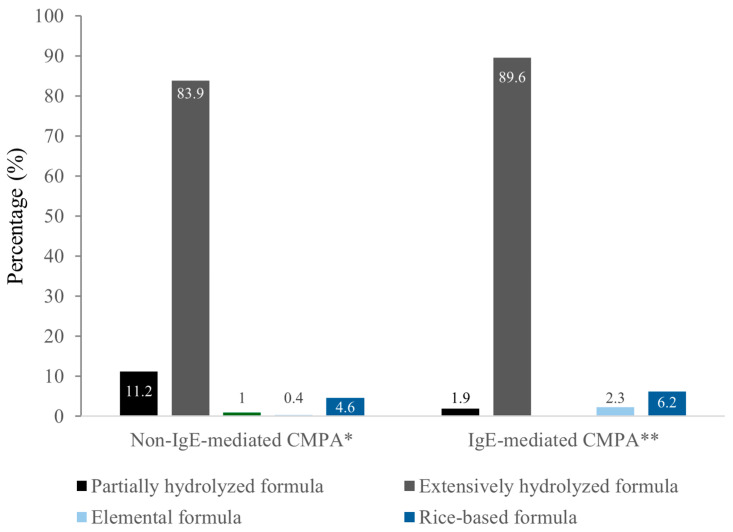
Preferred CMP-free diet formulas. Total number of valid responses: * N = 261; ** N = 259.

In non-IgE-mediated CMPA cases, clinicians most often prescribed eHF (83.9); corresponding figures for IgE mediated cases were 89.6% (Figure 3). Even so, partially hydrolyzed formulas were used by 11.2% and 1.9% of respondents in non-mediated and IgE mediated cases, respectively, and elemental, or rice-based formulas were less frequent in both phenotypes (Figure 3).

Perceptions regarding the potential use of alternative formulas as diagnostic elimination options were also explored: 98.1% of respondents “Agreed” and “Strongly Agreed” that HRF can be considered for diagnostic elimination, and 70.1% supported conditional use of soy based formulas for cultural, economic, or palatability reasons, though 20.3% still “disagreed” or “strongly disagreed” with this approach (Supplementary Table S9).

When analyzed by specialty, G/PA were more supportive of HRF as viable alternatives for diagnostic elimination diets (*p* = 0.015).

## 4. Discussion

Our findings align with international guidelines, which underscore the central role of detailed clinical history in diagnosis [5], with nearly all pediatricians (94.8%) reporting relying on it to establish a diagnostic suspicion. However, a quarter of respondents reported using symptom rating scales such as the Cow’s Milk-related Symptom Score (CoMiSS™), as part of their diagnostic methods, despite its status as an awareness tool rather than a diagnostic one. CoMiSS™, while widely studied, lacks standardization in cut-off values and presents considerable variability in sensitivity and specificity across different populations. The reported use of CoMiSS™ in diagnostic practice may reflect either a lack of clarity among professionals regarding its intended purpose, or the need for simplified tools in busy clinical settings [5,12,13,14,15].

After diagnostic suspicion, a phase of diagnostic elimination diet is implemented. Our data showed a tendency to maintain exclusion for 2 to 6 weeks, in line with guidelines suggesting at least 2–4 weeks to observe symptom resolution before the challenge [5]. Yet, variability in duration, especially in IgE-mediated CMPA, reflects the absence of uniform protocols and the need to individualize management according to severity and clinical response. Regarding CMP reintroduction for diagnostic confirmation, almost half of the participants reported they consistently performed OFC-D, which is considered the gold standard for confirming CMPA [16]. Nonetheless, a relatively low frequency of its systematic use was observed, particularly in IgE-mediated CMPA (10.8%). This finding also aligns with current ESPGHAN and DRACMA guidelines, which state that OFC-D is not mandatory when there is a clear clinical history of immediate reactions with strongly positive specific IgE, or in severe cases where reintroduction may pose unacceptable risks [5,9,17,18]. Reluctance to perform OFC-D in such scenarios may therefore reflect appropriate caution rather than deviation from best practice. Nonetheless, previous studies have shown variability. For instance, Vandenplas et al. (2023) [19] reported that 23% of caregivers declined an OFC-D when entering a randomized controlled trial, while Pérez et al. (2018) [11] observed that only around only 33% of the Spanish gastroenterologists considered OFC-D necessary to CMPA diagnosis [10]. In the absence of systematic OFC-D, many children may be diagnosed based solely on clinical suspicion or sensitization tests, which lack specificity, thereby increasing the risk of overdiagnosis in the real-world setting [19].

In addition to frequency of use, our study also evaluated the preferred settings for OFC-D. Current guidance specifies that while OFC-D can safely be performed at home in mild non-IgE-mediated suspected cases, it must be supervised in a hospital for IgE-mediated suspected forms, especially when there is a history of severe reactions [5,17,20,21]. In line with this, the majority of pediatricians supported home-based OFC-D in non-IgE suspected cases, whereas most reported hospital-based challenges in IgE suspected CMPA cases. An interesting finding is the variability between specialties in how OFC-D protocols are applied. In suspected IgE-mediated cases, G/PA specialists adhered more strictly to guideline-based schemes, whereas in non-IgE suspected CMPA cases, especially in breastfed or formula-fed infants, a relevant percentage favored their hospital-specific protocols, when compared to general pediatricians [11,16]. Indeed, one may speculate that G/PA specialists, given their higher exposure and expertise, are more cautious in high-risk scenarios such as IgE-mediated CMPA, while exercising more flexibility in low-risk, non-IgE cases. This interpretation is consistent with our results showing that gastroenterologists and PA received the highest proportion of referred cases, with PA more frequently managing the complex and IgE-mediated forms (69.3%). Conversely, general pediatricians may be more heterogeneous in their approaches due to differences in training and resource availability.

Tolerance acquisition monitoring also varied across our respondents. International guidelines recommend that the initial therapeutic elimination diet in CMPA should be maintained for a maximum of 6 months or until the infant reaches 12 months of age, whichever occurs first [5,17,18]. At that point, an OFC should be performed to assess the development of tolerance (OFC-T). In the case of IgE-mediated CMPA, it is also advised to monitor serum specific IgE levels prior to the OFC-T, in order to help guide its timing.

In the present study, about half of pediatricians reported reassessing tolerance every 3 to 6 months in non-IgE-mediated CMPA, which partially aligns with the above highlighted guidelines. However, a notable proportion reassessed either more frequently (15.6%) or less often (12.6%). This is consistent with previous findings from a survey of Spanish pediatric gastroenterologists, where only approximately 34% reported reassessing tolerance at the recommended 6-month intervals, and many extended follow-up beyond one year [11]. For IgE-mediated CMPA specifically, many pediatricians in our sample reported longer intervals (6–12 months) between reassessments, which could delay timely reintroduction. This is noteworthy given that reassessment schedules are often adapted in more severe cases or in infants with FPIES, where a longer elimination period is frequently maintained before attempting reintroduction [15,22]. Nonetheless, these patterns raise concerns: infrequent reassessment may lead to unnecessary prolongation of elimination diets, with potential nutritional and psychosocial implications; conversely, overly frequent reassessment may increase the risk of premature reintroduction and subsequent allergic reactions. Therefore, clear and stratified guidelines, tailored to disease severity, are essential to support appropriate reassessment intervals and avoid mismanagement of both mild and high-risk CMPA cases.

Home-based challenge (OFC-T) in mild cases of non-IgE-mediated CMPA was again widely accepted by the surveyed professionals. The majority of participants reported recommending the use of the milk ladder protocol, which is consistent with current evidence supporting the safety and efficacy of this stepwise reintroduction approach in such scenarios [23,24,25,26]. Interestingly, G/PA specialists were more likely to conduct pre-reintroduction sensitization testing and tended to use their own protocols, indicating a more informed and specialized approach. Moreover, while most participants overall preferred hospital-based settings for tolerance testing in patients with an unfavorable response to a previous OFC, G/PA specialties more frequently favored testing at home. Indeed, in our study, we observed significantly greater confidence in home-based challenges among G/PA specialists, likely supported by their more consistent provision of written instructions to families compared to general pediatricians. This suggests that confidence in home-based testing is closely tied to the amount and quality of guidance provided to caregivers, emphasizing the critical role of structured education in ensuring safe implementation. Regardless, this trend highlights the importance of reinforcing safety considerations and individualized risk assessment in all tolerance testing strategies.

Finally, dietary management in our study generally aligned with guidelines. eHF were the most commonly prescribed first-line treatment, consistent with ESPGHAN and DRACMA recommendations [5,9,18,27]. Soy formulas were rarely chosen, consistent with current restrictions due to cross-reactivity and phytoestrogen concerns [15,28]. The increasing acceptance of HRF reported in our cohort also reflects international data showing that up to 23–25% of pediatricians in some European countries now recommend HRF as an alternative in CMPA, particularly when eHF is poorly tolerated or unavailable [2,15,19]. However, it is concerning that almost 12% of pediatricians reported prescribing partially hydrolyzed formulas for CMPA, and nearly 2% even in IgE-mediated cases. Partially hydrolyzed formulas are not considered hypoallergenic and may still contain peptides capable of eliciting reactions, which is why current guidelines clearly state that they should not be used for the treatment of suspected or confirmed CMPA, particularly in IgE-mediated forms [29]. This represents a deviation from evidence-based recommendations and may expose children to persistent symptoms or adverse reactions.

Our findings reflect important parallels with international data, including reliance on clinical history, underuse of OFC in IgE-mediated cases, variability in elimination diet duration and tolerance monitoring, and diverse formula practices. Several factors may help explain the variability observed in diagnostic and management practices among Spanish pediatricians. Differences in training backgrounds, access to allergy or gastroenterology specialists, and the organizational structure of the healthcare setting—particularly the contrast between primary care and hospital-based environments—likely contribute to the heterogeneity in clinical decision-making. In addition, inconsistent dissemination and implementation of guideline updates may result in divergent interpretations of recommended approaches, especially regarding the duration of diagnostic elimination diets, the criteria for conducting oral food challenges, and the safety considerations when advising home-based reintroductions. These variations have relevant clinical implications: non-standardized diagnostic pathways may increase the risk of overdiagnosis, unnecessarily prolong CMP-free diets, or delay timely tolerance acquisition, while insufficiently supervised reintroduction strategies may compromise patient safety. Together, these findings underscore the importance of strengthening professional training, harmonizing protocols across care levels, and developing clearer, nationally coordinated pathways to ensure consistent and evidence-based management of CMPA.

This study has limitations, notably its reliance on self-reported practices, which may introduce recall or social desirability bias. Additionally, the predominance of urban-based respondents may reduce the generalizability of the findings to rural or less-resourced settings. Furthermore, although the survey captured information on specialty, workplace, and type of practice, it did not include variables such as years of professional experience or residency status, which could have provided valuable context for interpreting variability in diagnostic and management approaches.

Despite its limitations, this study presents several notable strengths. It is one of the few surveys to comprehensively capture real-world clinical practices in the diagnosis, management, and tolerance acquisition of CMPA across both primary and hospital pediatric care settings in Spain. The sample size was considerable, and the inclusion of a wide geographic distribution adds robustness to the findings. Moreover, the detailed intergroup analysis by specialty offers valuable insights into how professional background influences clinical decision-making, allowing for targeted improvements in guideline dissemination and educational interventions.

## 5. Conclusions

Overall, this study highlights substantial variability in CMPA management among Spanish pediatricians, particularly in oral food challenge use, exclusion diet duration, tolerance monitoring, and formula selection. These differences were influenced by clinician specialty. While many practices align with guidelines, inconsistencies may delay reintroduction or compromise safety. Future studies should address the standardization of procedures and the evaluation of long-term outcomes to optimize CMPA management in real-world practice.

## Figures and Tables

**Table 1:** OFC approaches to confirm diagnosis in infants with suspected CMPA.

For performing the home-based OFC in infants with suspected mild or moderate non-IgE-mediated CMPA who are fed with specialized infant formulas for CMPA how do you proceed?, *n* (%) *	
Following the current guidelines by indicating that it should be replaced by a CMP-containing standard formula.	236 (91.1)
Following a different protocol. Specify	23 (8.9)
For performing OFC in infants with suspected mild or moderate non-IgE-mediated CMPA, who are breastfed how do you proceed?, *n* (%) **	
Following the current guidelines by indicating that cow’s milk and dairy products should be reintroduced into the maternal diet	239 (90.5)
Following a different protocol. Specify	25 (9.5)
For performing OFC in infants with suspected IgE-mediated CMPA how do you proceed?, *n* (%) *	
Following the clinical guidelines by starting the challenge test with a very small dose, gradually increasing the volume	174 (67.2)
Following a different protocol. Specify	85 (32.8)

Total number of valid responses: * N = 259; ** N = 264.

**Table 2:** Pediatricians’ approaches to home tolerance testing.

For home tolerance testing in infants with mild to moderate non-IgE mediated CMPA how do you proceed?, *n* (%) *	
Following the staggered “milk ladder” strategy, according to guidelines.	252 (96.6)
Following the specific guidelines from your facility. Indicate	6 (2.3)
We have our own guidelines based on your personal experience. Indicate	3 (1.2)
To explain to parents how to perform the home tolerance test in infants with mild to moderate non-IgE mediated CMPA how do you proceed?, *n* (%) **	
Only explaining the instructions in the office	56 (21.3)
Complementing the explanations in the office with information material on paper, with the instructions to be followed	194 (73.8)
Supplementing the explanations in the office with online information material, with the instructions to follow	13 (4.9)
In patients with mild or moderate non-IgE-mediated CMPA, with a previous unfavorable response to CMP reintroduction, where do you prefer to perform new tolerance acquisition tests?, *n* (%) ***	
At home	72 (27.9)
In the hospital/Under medical supervision	186 (72.1)

Total number of valid responses: * N = 262; ** N = 263; *** N = 259.

## Data Availability

The data presented in this study are available on request from the corresponding author.

## Supplementary Materials

The following supporting information can be downloaded at https://www.mdpi.com/article/10.3390/children12121645/s1: Figure S1: (A) Recommended duration of CMP-free diet after a CMPA diagnosis, (B) Minimum age recommended to maintain a CMP-Free diet after a CMPA diagnosis, (C) Requirement of IgE/skin prick testing before CMP reintroduction; Table S1: Sociodemographic characteristics of the study participants; Table S2: Diagnostic practices and clinical distribution of CMPA types; Table S3: Diagnostic practices and CMPA management strategies by age and specialty; Table S4: Symptom profiles used to suspect CMPA; Table S5: Duration of CMP elimination diet and reintroduction strategies; Table S6: Physicians’ recommendations on introducing new foods during diagnostic challenges; Table S7: Referral practices following CMPA diagnosis; Table S8: Safety perceptions of home tolerance testing in mild to moderate non IgE-mediated CMPA; Table S9: Pediatricians’ perceptions regarding the use of rice- and soy-based infant formulas in CMPA management.

## Abbreviations

The following abbreviations are used in this manuscript:

AAF	Amino Acid Formula
CMP	Cow’s Milk Protein
CMPA	Cow’s Milk Protein Allergy
CoMiSS™	Cow’s Milk-related Symptom Score
DRACMA	Diagnosis and Rationale for Action against Cow’s Milk Allergy
eHF	Extensively Hydrolyzed Formula
ETAPA	Survey on Tolerance Acquisition in Cow’s Milk Protein Allergy in Primary and Hospital Pediatric Care
ESPGHAN	European Society for Pediatric Gastroenterology, Hepatology and Nutrition
FPIES	Food Protein-Induced Enterocolitis Syndrome
G/PA	Gastroenterologists and Pediatric Allergologists
HRF	Hydrolyzed Rice Formula
IgE	Immunoglobulin E
OFC	Oral Food Challenge
OFC-D	Diagnostic Oral Food Challenge
OFC-T	Tolerance Oral Food Challenge
PA	Pediatric Allergologist
SD	Standard Deviation
SPSS	Statistical Package for the Social Sciences

## Appendix A

### Appendix A.1. Clinical Practice Questionnaire

#### Appendix A.1.1. A. Respondent Profile

**1. Gender** ▢ Female ▢ Male **2. Age:** ______ years **3. Province of practice:** __________________ **4. Work setting (choose one):** ▢ Public ▢ Private ▢ Both **5. Location of workplace:** ▢ Rural (<2000 inhabitants) ▢ Semi-urban (2000–10,000) ▢ Urban (>10,000) **6. Type of facility (check all that apply):** ▢ Primary care ▢ Public hospital ▢ Private consultation ▢ Private hospital ▢ Other: __________ **7. Specialty:** ▢ General pediatrics ▢ Pediatric gastroenterology ▢ Pediatric allergology ▢ Family doctor working as pediatrician ▢ Other: __________

#### Appendix A.1.2. B. CMPA Diagnosis

**8. In your work setting, is it common to refer CMPA cases to a specialist, or are they monitored exclusively in primary care?** Select only one option: ▢ Referred to specialist ▢ Managed in primary care ▢ Depends on case **9. If referred to a specialist, to whom do you refer each type of allergy?** **IgE-mediated:** ▢ Gastroenterology ▢ Allergy ▢ Other **Non-IgE-mediated:** ▢ Gastroenterology ▢ Allergy ▢ Other **10. To establish a diagnostic suspicion of CMPA, do you conduct a detailed clinical history including physical exam, nutritional assessment, and dietary history?** ▢ Yes, always ▢ Yes, sometimes ▢ Occasionally ▢ Almost never ▢ Never **11. Which of the following methods do you use for diagnosing CMPA? (Check all that apply):** ▢ Symptom scoring ▢ Allergen elimination ▢ Prick test ▢ Specific IgE testing ▢ Challenge tests **12. Indicate the symptoms you associate with suspected CMPA (select all that apply):**		
**Symptoms**	**IgE-mediated CMPA**	**Non-IgE-mediated CMPA**
Anaphylaxis	▢	▢
Iron-deficiency anemia	▢	▢
Angioedema	▢	▢
Asthma	▢	▢
Colic, irritability	▢	▢
Conjunctivitis	▢	▢
Diarrhea	▢	▢
Dysphagia	▢	▢
Mild hoarseness	▢	▢
Eczema (atopic dermatitis)	▢	▢
Perianal rash	▢	▢
Constipation	▢	▢
Anal fissures	▢	▢
Lack of improvement	▢	▢
Bottle/food refusal	▢	▢
Regurgitation, vomiting	▢	▢
Rhinitis	▢	▢
Blood in stools	▢	▢
Wheezing	▢	▢
Oral allergy syndrome	▢	▢
Chronic cough	▢	▢
Acute urticaria	▢	▢
**13. Of all the CMPA cases with suspected diagnosis in a year, approximately what percentage are:** **Non-IgE-mediated:** ___% Mild/moderate: ___% Severe: ___% **IgE-mediated:** ___% Mild/moderate: ___% Severe: ___% **14. How long do you exclude cow’s milk proteins (CMP) from the diet for diagnostic purposes?** **If non-IgE-mediated CMPA is suspected** ▢ A period not exceeding 2 weeks ▢ A period not exceeding 4 weeks ▢ A period not exceeding 6 weeks ▢ A period not exceeding 8 weeks ▢ Other. Specify: ___________ **If IgE-mediated CMPA is suspected** ▢ A period not exceeding 2 weeks ▢ A period not exceeding 4 weeks ▢ A period not exceeding 6 weeks ▢ A period not exceeding 8 weeks ▢ Other. Specify: ___________ **15. If symptoms resolve after CMP elimination, do you reintroduce CMP in a controlled way (diagnostic challenge), except in severe FPIES cases, to confirm CMPA diagnosis?** ▢ Yes, always ▢ Yes, sometimes ▢ Occasionally ▢ Almost never ▢ Never **In suspected IgE-mediated CMPA** ▢ Yes, always ▢ Yes, sometimes ▢ Occasionally ▢ Almost never ▢ Never **16. For mild/moderate suspected CMPA, where do you prefer the diagnostic challenge to be conducted?** **Non-IgE-mediated CMPA** ▢ At Home ▢ In hospital/under medical supervision **IgE-mediated CMPA** ▢ At Home ▢ In hospital/under medical supervision **17. For at-home challenge tests in infants with suspected mild/moderate non-IgE-mediated CMPA fed with special formula:** ▢ You instruct to replace one scoop of special formula per day with one of standard formula in at least two feedings, and if no symptoms appear, increase gradually until full reintroduction. ▢ You follow another protocol. Specify: ___________ **18. For conducting an at-home challenge test in infants with suspected mild/moderate non-IgE-mediated CMPA who are breastfed:** ▢ You advise reintroducing cow’s milk and dairy into the mother’s diet, starting with 1 serving of milk or dairy per day during the first week, progressively increasing the amount, and monitoring for possible symptom recurrence up to 4 weeks after reintroduction ▢ You follow another protocol. Specify: ___________ **19. For performing a challenge test in infants with suspected IgE-mediated CMPA:** ▢ Begin the challenge with a very small dose, gradually increasing volume up to at least 100 mL, monitor the patient for at least 2 h after the final dose. If no reaction occurs, cow’s milk should be continued at home daily at a minimum of 200 mL/day for at least 2 weeks. ▢ You follow another protocol. Specify: ___________ **20. Do you remind families not to introduce any new foods into the diet during the challenge test?** ▢ Yes, always ▢ Yes, sometimes ▢ Occasionally ▢ Almost never ▢ Never		

#### Appendix A.1.3. C. CMPA Follow-Up

**21. In cases of mild/moderate non-IgE-mediated CMPA, how long do you maintain a CMP-free diet?** ▢ < 1 month ▢ 1–3 months ▢ 3–6 months ▢ 6–12 months ▢ > 12 months **22. In cases of IgE-mediated CMPA, how long do you maintain a CMP-free diet?** ▢ < 1 month ▢ 1–3 months ▢ 3–6 months ▢ 6–12 months ▢ > 12 months **23. What is the minimum age at which you believe a CMP-free diet should be maintained?** ▢ 6 months ▢ 9 months ▢ 12 months ▢ 15 months ▢ 18 months ▢ 24 months ▢ Depends on phenotype or severity **24. Before reintroducing cow’s milk proteins (CMP), do you consider it necessary to perform specific IgE testing and/or a skin prick test?** **For non-IgE-mediated CMPA:** ▢ Yes, always ▢ Yes, sometimes ▢ Occasionally ▢ Almost never ▢ Never **For IgE-mediated CMPA:** ▢ Yes, always ▢ Yes, sometimes ▢ Occasionally ▢ Almost never ▢ Never **25. How often do you assess the development of tolerance?** **For non-IgE-mediated CMPA:** ▢ Monthly ▢ Every 1–3 months ▢ Every 3–6 months ▢ Every 6–12 months ▢ Depends on the case ▢ Other. Specify: ___________ **For IgE-mediated CMPA:** ▢ Monthly ▢ Every 1–3 months ▢ Every 3–6 months ▢ Every 6–12 months ▢ Depends on the case ▢ Other. Specify: ___________ **26. For conducting an at-home tolerance test in infants with mild/moderate non-IgE-mediated CMPA:** ▢ Follow the stepped approach of the “milk ladder”: start with foods containing baked/cooked milk, then progress through dairy products (yogurt, cheese), and finish with direct milk intake. ▢ Follow the protocol established at your workplace. Specify: ___________ ▢ Follow your own protocol based on personal experience. Specify: ___________ **27. When explaining to parents how to conduct the at-home tolerance test in infants with mild/moderate non-IgE-mediated CMPA:** ▢ You only give verbal instructions during the consultation ▢ You supplement the consultation with printed instructions ▢ You supplement the consultation with online instructional materials **28. Do you consider that, following appropriate guidelines and recommendations, at-home tolerance tests for mild/moderate non-IgE-mediated CMPA are safe?** ▢ Yes, completely ▢ Yes, mostly ▢ It depends on the case ▢ No, mostly (specify): ___________ ▢ No, not at all (specify): ___________ **29. In patients with mild/moderate non-IgE-mediated CMPA and a previous negative response to CMP reintroduction, where do you prefer to conduct new tolerance acquisition tests?** ▢ At home (specify): ___________ ▢ In hospital / under medical supervision (specify): ___________ **30. Of all the tolerance tests performed on your CMPA patients, what percentage are successful and confirm resolution of CMPA?** ___ % **31. Indicate your level of agreement with the following statements regarding infant formulas based on rice or soy protein:** (a) “Although less studied than extensively hydrolyzed formulas based on cow’s milk, hydrolyzed rice formulas may be considered an alternative for a diagnostic elimination diet.” ▢ Strongly agree ▢ Agree ▢ Neutral ▢ Disagree ▢ Strongly disagree (b) “Soy-based infant formula should not be used as the first choice for a diagnostic elimination diet, but it may be considered in certain cases for economic, cultural, or palatability reasons.” ▢ Strongly agree ▢ Agree ▢ Neutral ▢ Disagree ▢ Strongly disagree **32. When prescribing a CMP-free diet, I prefer to start with:** **In suspected non-IgE-mediated CMPA:** ▢ Partially hydrolyzed formula ▢ Extensively hydrolyzed formula ▢ Elemental formula ▢ Soy-based formula ▢ Rice-based formula **In suspected IgE-mediated CMPA:** ▢ Partially hydrolyzed formula ▢ Extensively hydrolyzed formula ▢ Elemental formula ▢ Soy-based formula ▢ Rice-based formula
