# Supplementary material for: Cow’s Milk Protein Allergy: ETAPA Survey on Pediatric Management and Tolerance Acquisition
Source: Children (Basel). 2025 Dec 3;12(12):1645. doi: 10.3390/children12121645 (PMC12731325; doi:10.3390/children12121645)
Supplement: Supplementary file 1 [file children-12-01645-s001.zip › Supplementary Material_06112025.pdf]

Cow's Milk Protein Allergy: ETAPA Survey on Pediatric management and tolerance acquisition

Supplementary Material

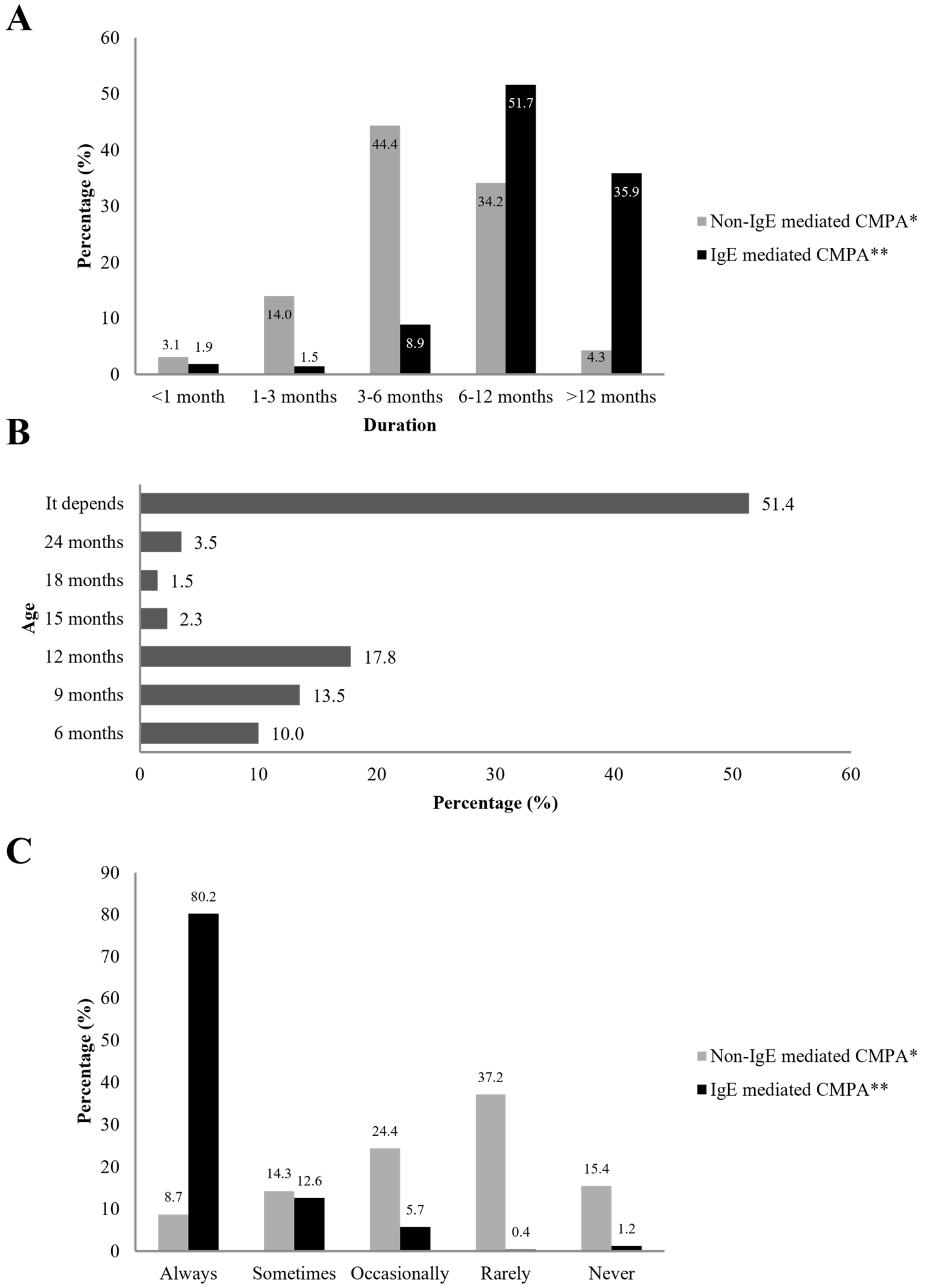

Supplementary Figure S1. Recommended duration of CMP-free diet after a CMPA diagnosis. Total number of valid responses: *N=257; **N=259. (B) Minimum Age Recommended to

Maintain a CMP-Free Diet after a CMPA diagnosis*. Total number of valid responses: *N=259. (C) Requirement of IgE/skin prick testing before CMP reintroduction. Total number of valid responses: *N=266; **N=262

Supplementary Table S1. Sociodemographic characteristics of the study participants.

	**Pediatricians (N=269)**
**Age (years old), mean (SD)***	48.3 (11.0)
**Sex, n (%)*****	
Female	165 (62.3)
Male	100 (37.7)
**Work setting, n (%)***	
Public	158 (60.1)
Private	52 (19.8)
Both	53 (20.2)
**Location of the workplace, n (%)****	
Urban	230 (87.8)
Semi-urban	28 (10.7)
Rural	4 (1.5)
**Type of facility**	
Primary Care	149 (55.4)
Public Hospital	54 (20.1)
Private Practice	48 (17.8)
Private Hospital	18 (6.7)
Other	12 (4.5)
**Specialty****	
General Pediatrics	187 (71.4)
Pediatric Gastroenterology	50 (19.1)
Pediatric Allergology	11 (4.2)
Family Doctor working as pediatrician	7 (2.7)
Others	7 (2.7)

Total number of valid responses: *N=263; **N=262; ***N=265

Supplementary Table S2. Diagnostic practices and clinical distribution of CMPA types

**To establish a diagnostic suspicion of CMPA, do you perform a detailed medical history, including physical examination, nutritional assessment, and dietary history?, n (%)***	
Yes, always	254 (94.8)
Yes, sometimes	11 (4.1)
Occasionally	3 (1.1)
**Which of the following methods do you use in the diagnosis of CMPA?, n (%)**	
Allergen Elimination	111 (41.3)
Specific IgE Determination	75 (27.9)
Oral Food Challenge	71 (26.4)
Symptom Assessment Scale	68 (25.3)
Prick Test	34 (12.6)

Total number of valid responses: *N=268

Supplementary Table S3. Diagnostic practices and CMPA management strategies by age and specialty.

	Specialty
	Other **(G/PA)**
**CMPA cases referral to a specialist:**	**0.00000**
IgE-mediated	0.38230
Non-IgE mediated	0.40340
**Most common methods used in the diagnosis of CMPA:**	
Symptom Assessment Scale	0.31529
Allergen Elimination	0.18070
Prick Test	**0.04771**
Specific IgE Determination	***0.00316***
Challenge Tests	**0.02064**
**Performance of a detailed medical history (including physical examination. nutritional assessment. etc) to establish a diagnostic suspicion of CMPA**	0.85720
**Approximate % of non-IgE-mediated CMPA cases identified of all CMPA cases with a diagnostic suspicion over one year**	0.69611
Mild/Moderate cases	0.24066
Severe cases	0.24066
**Approximate % of IgE-mediated CMPA cases identified of all CMPA cases with a diagnostic suspicion over one year**	0.23308
Mild/Moderate cases	0.43511
Severe cases	0.43511
**CMP exclusion time required to establish a diagnosis**	
In on-IgE-mediated CMPA	0.31850
In IgE-mediated CMPA	0.12700
**Decision to always reintroduce CMP in a controlled manner (OFC) if symptoms resolve after the exclusion period of CMP from the diet**	
In non-IgE-mediated CMPA	***0.00670***
In cases of suspected IgE-mediated CMPA	0.05290
**Preference for home-based OFC**	
In cases of suspected mild to moderate non-IgE-mediated CMPA	0.57692
In cases of suspected mild to moderate IgE-mediated CMPA	0.27624
**Preference in following current guidelines when performing home-based OFC**	
In formula-fed infants with suspected mild/moderate non-IgE-mediated CMPA	***0.00105***
In breastfed infants with suspected mild/moderate non-IgE-mediated CMPA	***0.04427***
In infants with suspected IgE-mediated CMPA	**0.01072**
**Reminder of families that no new foods should be introduced into the diet while OFC is being conducted**?	0.22100
**Preference for shorter CMP-free diet durations**	
In mild to moderate non-IgE-mediated CMPA	0.18530
In mild to moderate IgE-mediated CMPA	0.13240
**Preference for maintaining a CMP-free diet until older ages**	0.26660
**Preference in performing specific IgE and/or a prick testing before reintroducing CMP, following diagnosis:**	
In cases of suspected non-IgE-mediated CMPA	**0.00460**
In cases of suspected IgE-mediated CMPA	**0.03550**
**Frequency of tolerance acquisition assessment.**	
In cases of suspected non-IgE-mediated CMPA	0.11730
In cases of suspected IgE-mediated CMPA	0.32040
**Preference for their own protocols in home tolerance testing of infants with mild to moderate non-IgE-mediated CMPA**	**0.00800**
**Preference for supplementing parental guidance on home-based tolerance testing with printed instructional materials.**	**0.01310**
**Recognition of home tolerance testing in patients with mild or moderate non-IgE mediated CMPA as totally safe**	**0.03070**
**Preference for home-based tolerance tests in mild to moderate non-IgE mediated CMPA with prior unfavorable response to CMP reintroduction.**	**0.00004**
**% of tolerance tests considered successful at confirming CMPA resolution**[a]	0.20844

**Recognition of HRF as an alternative for a diagnostic elimination diet**	**0.01500**
**Recognizing the use of soy infant formula in specific cases (e.g., economic)**	0.42820

P values highlighted in **bold** and ***italic*** in favor of other specialties.
P values highlighted in **bold** in favor of Gastroenterology or Pediatric Allergology (G/PA).
Statistical significance was obtained through Fishers Test analysis unless indicated otherwise.
[a]Significance obtained through T-test/Anova analysis.

Supplementary Table S4. Symptom profiles used to suspect CMPA

**Symptoms Considered Related to CMPA Suspicion**		
**Items**	**Non-IgE-mediated n (%)**	**IgE-Mediated n (%)**
Anaphylaxis	7 (2.6)	249 (92.6)
Iron-deficiency anemia	193 (71.8)	25 (9.3)
Angioedema	8 (3.0)	236 (87.7)
Asthma	18 (6.7)	199 (74.0)
Colic, irritability	248 (92.2)	53 (19.7)
Conjunctivitis	20 (7.4)	107 (39.8)
Diarrhea	236 (87.7)	105 (39.0)
Dysphagia	122 (45.4)	83 (30.9)
Mild dysphonia	49 (18.2)	104 (38.7)
Eczema (atopic dermatitis)	105 (39.0)	196 (72.9)
Perianal rash	155 (57.6)	65 (24.2)
Constipation	226 (84.0)	31 (11.5)
Anal fissures	165 (61.3)	17 (6.3)
Lack of improvement	150 (55.8)	72 (26.8)
Feeding/bottle refusal	213 (79.2)	135 (50.2)
Regurgitation, vomiting	240 (89.2)	121 (45.0)
Rhinitis	21 (7.8)	143 (53.2)
Blood in stool	211 (78.4)	80 (29.7)
Wheezing	17 (6.3)	199 (74.0)
Oral allergy syndrome	22 (8.2)	191 (71.0)
Chronic cough	56 (20.8)	108 (40.2)
Acute urticaria	17 (6.3)	238 (88.5)

Supplementary Table S5. Duration of CMP elimination diet and reintroduction strategies

**To establish a diagnosis of CMPA, for how long do you exclude CMP from the diet?, n (%)**	**Non-IgE-mediated CMPA*:**	**IgE-mediated CMPA**:**
A period not exceeding 2 weeks	37 (14.0)	47 (18.0)
A period not exceeding 4 weeks	117 (44.3)	60 (23.0)
A period not exceeding 6 weeks	62 (23.5)	25 (9.6)
A period not exceeding 8 weeks	35 (13.3)	31 (11.9)
Other	13 (4.9)	98 (37.6)
**If, after the exclusion period of CMP from the diet, symptoms resolve, do you reintroduce CMP in a controlled manner (OFC), except in cases of severe FPIES, to confirm the diagnosis of CMPA?, n (%)**	**Non-IgE-mediated CMPA**:**	**IgE-mediated CMPA***:**
Yes, always	127 (48.7)	27 (10.8)
Yes, sometimes	76 (29.1)	34 (13.6)
Occasionally	22 (8.4)	37 (14.7)
No, rarely	29 (11.1)	65 (25.9)
No, never	7 (2.7)	88 (35.1)

Total number of valid responses: *N=263; **N=261; ***N=251

Supplementary Table S6. Physicians' recommendations on introducing new foods during diagnostic challenges

**Do you remind families that no new foods should be introduced into the diet while the diagnostic challenge is being conducted?, n (%)***	
Yes, always	238 (89.5)
Yes, sometimes	17 (6.4)
Occasionally	6 (2.3)
No, rarely	2 (0.8)
No, never	3 (1.1)

Total number of valid responses: *N=266

Supplementary Table S7. Referral practices following CMPA diagnosis

**Is it common to refer CMPA cases to a specialist, or are they managed exclusively in primary care?, n (%)***		
They are referred to a specialist	94 (35.5)	
They are managed in primary care	39 (14.7)	
It depends on the case	132 (49.8)	
**In the case of referral to a specialist, to whom is each type of allergy referred?, n (%)**	**Non-IgE-mediated***	**IgE-mediated****
Gastroenterology	219 (92.4)	73 (30.3)
Allergology	16 (6.8)	167 (69.3)
Others	2 (0.8)	1 (0.4)
**Of all CMPA cases with a diagnostic suspicion over one year, what is the approximate percentage of the following, % (SD)**	**Non-IgE-mediated CMPA**	**IgE-mediated CMPA**
	70.2 (23.0)	27.3 (21.1)
Mild/Moderate cases	85.3 (10.5)	80.4 (19.8)
Severe cases	14.7 (10.5)	19.6 (19.8)

Total of valid responses: *N=265;**N=241; ***N=237

Supplementary Table S8. Safety perceptions of home tolerance testing in mild to moderate non-IgE-mediated CMPA

**Do you consider that, following the appropriate guidelines and recommendations, home tolerance testing in patients with mild or moderate non-IgE mediated CMPA is safe?, n (%)***	
Yes, totally	67 (25.1)
Yes, mostly	151 (56.6)
Depends on the case	46 (17.2)
No, mostly (specify)	2 (0.8)
No, not at all (specify)	1 (0.4)

Total number of valid responses: *N=267

Supplementary Table S9. Pediatricians' perceptions regarding the use of rice- and soy-based infant formulas in CMPA management

**Please rate your degree of agreement with the following statements about infant formulas based on rice or soy proteins, n (%)**	
*Although less studied than eHF based on cow's milk, HRF can be considered as an alternative for a diagnostic elimination diet**	
Strongly disagree	0 (0.0)
Disagree	3 (1.2)
Indifferent	2 (0.8)
Agree	136 (52.5)
Strongly agree	118 (45.6)
*Soy infant formula should not be used as the first choice for diagnostic elimination diet, but may be considered in some cases for economic, cultural, and palatability reasons***	
Strongly disagree	14 (5.4)
Disagree	39 (14.9)
Indifferent	25 (9.6)
Agree	132 (50.6)
Strongly agree	51 (19.5)

Total number of valid responses: *N=259; **N=261
